# Centuries of monthly and 3-hourly global ocean wave data for past, present, and future climate research

**DOI:** 10.1038/s41597-020-0566-8

**Published:** 2020-07-10

**Authors:** Zhenya Song, Ying Bao, Danqi Zhang, Qi Shu, Yajuan Song, Fangli Qiao

**Affiliations:** 1 First Institute of Oceanography, Ministry of Natural Resources, Qingdao, 266061 China; 2 Laboratory for Regional Oceanography and Numerical Modeling, Pilot National Laboratory for Marine Science and Technology, Qingdao, 266237 China; 3 Key Laboratory of Marine Science and Numerical Modeling, Ministry of Natural Resources, Qingdao, 266061 China

**Keywords:** Physical oceanography, Climate and Earth system modelling

## Abstract

Ocean surface waves are essential to navigation safety, coastal activities, and climate systems. Numerical simulations are still the primary methods used in wave climate research, especially in future climate change scenarios. Recently, First Institute of Oceanography-Earth System Model version 2.0 (FIO-ESM v2.0), a global climate model coupled with an ocean wave model, was carried out the Coupled Model Intercomparison Project phase 6 (CMIP6) experiments. Here, we present the global monthly-mean and 3-hourly instantaneous wave parameter dataset from the FIO-ESM v2.0 CMIP6 experiments, including 700-year piControl, 165-year historical, three 86-year future scenarios (ssp125, ssp245, and ssp585, respectively), and two 150-year climate sensitive experiments (1pctCO2 and abrupt-4xCO2) simulations. Historical results show that the model can capture the basic wave climate features under climate change. These unique centuries of global wave data are from a fully coupled system and can provide the community with a vital long-term data source for scientific and engineering applications, such as wave climate research, wave-related process studies and parameterizations, as well as coastal and near-shore industry designs.

## Abstract (summary)

**Table:** 

Measurement(s)	spectrum peak wave period • zero-crossing wave period • wave • height • direction
Technology Type(s)	computational modeling technique
Factor Type(s)	time-variant • geographic location
Sample Characteristic - Environment	climate system • ocean
Sample Characteristic - Location	Earth (planet)

Machine-accessible metadata file describing the reported data: 10.6084/m9.figshare.12479582

## Background & Summary

Ocean surface waves (hereafter called ocean waves) are a kind of motion occurring on ocean and sea surfaces driven by surface winds. Ocean waves can travel thousands of miles with heights ranging from several centimeters to tens of meters before reaching land and vanishing. Therefore, ocean waves are valuable for navigation safety, coastal ecosystems, and offshore human activities and even play a crucial role in global and regional climate systems^1,2^.

A long-term dataset with high spatial resolution and temporal continuity is necessary for climate research. Although the number and coverage of *in situ* observations have been increasing (e.g., ICOADS)^3^, ocean wave data are still sparse in space and discontinuous in time. There has been nearly continuous coverage of global observations from satellite altimeters since 1985^4,5^, but this coverage only meets the time requirements for climate research and only provides the significant wave height. Therefore, numerical simulations are still the primary method to be used in ocean wave climate research, especially in future climate change scenarios.

For historical ocean wave climate research, several global hindcast/reanalysis datasets, such as the ERA series (e.g., ERA-Interim from 1979 to 2019^6^; ERA5 from 1979 to present^7^; ERA-20C from 1900 to 2010^8^; CERA-20C from 1901 to 2010^9^), EMC/NCEP 30-Year wave hindcast data from 1979 to 2009^10^, GFDL Wave Hindcast from 1981 to 2009^11^, Ifremer Wave Hindcast from 1990 to present^12^, CAWCR Wave Hindcast from 1979 to present^13^, JAR-55-Wave from 1958–2012^14^, and other similar datasets, have been carried out worldwide.

However, ocean waves are not included in most of the state-of-the-art global climate models, which is the key tool to assess and provide future projections of climate systems. Therefore, as the growing demand to understand the response of the global wave climate to increasing greenhouse gas concentrations, especially through the Coordinated Ocean Wave Climate Project (COWCLIP)^1^, several studies on future ocean wave climate research have provided ocean wave information by using the output of global and regional climate models to force the standalone ocean surface wave model^15–28^.

Recently, the First Institute of Oceanography-Earth System Model version 2.0 (FIO-ESM v2.0)^29^, a global climate model coupled with the ocean wave model through the wave-induced vertical mixing, the effects of Stokes drifts and sea spray on the air-sea flux, was used to carry out the Coupled Model Intercomparison Project phase 6 (CMIP6) experiments^30^. FIO-ESM v2.0 was integrated for 1000 years under pre-industrial conditions (piControl experiment), and the model reached a quasi-equilibrium state after 300 years. Then, FIO-ESM v2.0 conducted the historical simulation (AD 1850–2014), three future scenario experiments (ssp126, ssp245, and ssp585 covering AD 2015–2100), and two 150-year climate sensitive experiments (1pctCO2, and abrupt-4xCO2). The preliminary results showed that FIO-ESM v2.0 could capture the basic features of the ocean waves and the climate system^29^.

Here, we provide the monthly mean and 3-hourly instantaneous wave parameters, including significant wave height (Hs), mean wave direction (Dm), spectrum peak wave period (Tp), and zero-crossing wave period (Tz) from the FIO-ESM v2.0 CMIP6 experiments. As the wave model is one of the components of FIO-ESM v2.0, the wave statistics from the FIO-ESM v2.0 CMIP6 experimental data are unique in multiple scientific and engineering applications, which is different from previous ocean wave datasets. The wave data over 1000 years can contribute to wave climate research, such as improving our scientific understanding of climate variabilities, long-term trends, extremes, and scenario studies. Moreover, these data can also contribute to wave-related process studies and parameterizations, as well as coastal and near-shore industry designs, etc.

## Methods

In this section, we introduce the framework and configurations of FIO-ESM v2.0 and the design of the related CMIP6 experiments used in this study. FIO-ESM v2.0 is the global earth system model, which contains two parts: a coupled physical climate model and a carbon cycle model. As we did not consider the biogeochemical processes and only integrated part of the coupled physical climate model, FIO-ESM v2.0 is referred to as the coupled physical climate model in this study.

### FIO-ESM v2.0 and configuration

**Fig. 1:**
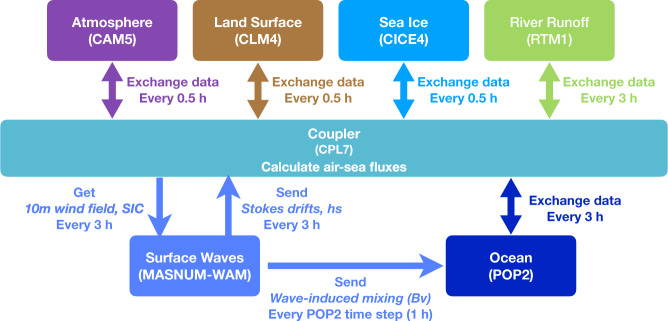
The framework and flowchart of FIO-ESM v2.0.

Despite the carbon cycle model components, FIO-ESM v2.0 is a global climate model consisting of the atmosphere, land surface, river runoff, sea ice, ocean, and ocean wave model components, which are connected through a coupler (Fig. 1). The components include the Community Atmosphere Model version 5 (CAM5)^31^, the Community Land Surface Model version 4.0 (CLM4.0)^32^, the River Transport Model (RTM)^33^, the Los Alamos National Laboratory sea ice model version 4 (CICE4)^34^, the Parallel Ocean Program version 2 (POP2)^35^, and the MArine Science and NUmerical Modeling (MASNUM) wavenumber spectrum wave model (MASNUM-WAM)^36^.

There are three distinctive physical processes related to ocean waves in FIO-ESM v2.0: (1) nonbreaking surface wave-induced vertical mixing (Bv)^37^, which has been incorporated into both FIO-ESM v1.0^38^ and FIO-ESM v2.0^29^, (2) Stokes drift, which influences the air-sea relative speed in the calculation of air-sea fluxes, and (3) sea spray, which could influence the air-sea heat fluxes. Bv can dramatically improve the upper ocean temperature, salinity and current simulation ability, and the other two physical processes are incorporated into FIO-ESM to physically improve the air-sea flux parameterization for the first time. Further information on FIO-ESM v2.0 can be found in Bao *et al*.^29^.

In this work, the resolution configuration is referred to as f09_gx1v6. The horizontal resolutions are f09 with a finite volume grid (approximately 0.9° × 1.25°) for both CAM5 (with 30 vertical layers) and CLM4.0 and a nominal 1° with the northern pole displaced into Greenland for POP2 (with 61 vertical layers), CICE4 and MASNUM-WAM. The actual horizontal resolution of nominal 1° is 1.125° in longitude and varies from 0.27° (at the equator) to 0.54° (far northwest Pacific) in latitude. In the wavenumber space of MASNUM-WAM, the angular resolution is 30°, and the wavenumber grid is adopted as follows:

$$K\left(i\right)={K}_{min}\,{\rm{\exp }}\left((i-1)\Delta K\right){\rm{,}}\,i=i,\,\ldots ,\,\ldots ,\,N+1$$

where

$${K}_{min}=0.0071,\,{K}_{max}=0.6894$$

$$\Delta K=\frac{1}{N}ln\frac{{K}_{max}}{{K}_{min}},\,N=25$$

The atmosphere, land surface, and sea ice component models exchange data with the coupler every 0.5 h, while the river runoff, ocean and wave models exchange data with the coupler at 3 h intervals. The MASNUM-WAM obtains wind field at 10 m height of ocean surface and sea ice concentration (SIC) from the coupler, is integrated to produce the wavenumber spectrum, and then calculates the nonbreaking surface wave-induced vertical mixing and other necessary variables for including the effects of Stokes drift and sea spray on air-sea flux, and finally sends Bv to POP2 and other variables to the coupler (Fig. 1). SIC is used to check whether the grid is covered by sea ice (sea ice concentration greater than 30%), where the wave spectrum is set to be zero. The configuration information of FIO-ESM v2.0 is summarized in Table 1.

**Table 1:** Configurations of FIO-ESM v2.0.

Component	Model version	Resolution	Coupling intervals
Atmosphere	CAM5	- H: f09 (0.9° × 1.25°) - V: 30 layers	0.5 h
Land surface	CLM4.0	- H: f09 (0.9° × 1.25°)	0.5 h
River runoff	RTM	- H: 0.5° × 0.5°	3 h
Sea ice	CICE4	- H: gx1v6 (1.125° × 0.27~0.54°)	0.5 h
Ocean	POP2	- H: gx1v6 (1.125° × 0.27~0.54°) - V: 61 layers, with the first layer is at 0 m for sea surface temperature (SST) diagnosed by the SST diurnal cycle parameterization.	3 h
Ocean wave	MASNUM-WAM	- H: gx1v6 (1.125° × 0.27~0.54°) - Wavenumber: 25 - Angle: 30°	3 h
Coupler	CPL7		

### Related CMIP6 experiment setup

**Fig. 2:**
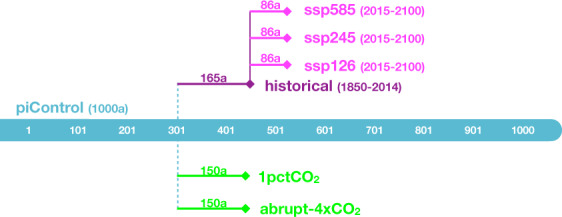
Diagram of CMIP6 experiments carried out by FIO-ESM v2.0 in this work.

Following the CMIP6 protocols^30^, FIO-ESM v2.0 was conducted with the Diagnostic, Evaluation and Characterization of Klima (DECK), a historical simulation, and six CMIP-Endorsed Model Intercomparison Projects (MIPs) for participating CMIP6^29^. As this dataset is used for wave climate research, we selected the wave output of the experiments related to the wave climate (Fig. 2, Table 2), including piControl, historical simulation, three future scenario experiments (ssp126, ssp245, and ssp585), and two climate sensitive experiments (1pctCO2, and abrupt-4xCO2). The CMIP6 forcing data are available from https://esgf-node.llnl.gov/search/input4mips/. The details of the experiments are summarized in Table 2.

**Table 2:** CMIP6 experiments carried out by FIO-ESM v2.0 in this work.

Experiment name	Number of simulated years	Experiment description	Initial condition
piControl	1000 a	- Preindustrial control simulation - Under conditions chosen as representative of the period to the onset of large-scale industrialization, with 1850 being the reference year	Integrated from observations
historical	165 a (AD 1850–2014)	- Historical simulation - Defined to begin in 1850 and end in 2014, forced by the evolving forcing based on observations provided by CMIP6	Branched from Jan 1^st^ 301a in piControl
**Future scenario experiments**			
ssp126	86 a (AD 2015–2100)	- Shared socioeconomic pathways (SSP1-2.6) - The low end of the range of future forcing pathways, in which the radiative forcing reaches 2.6 W m^−2^ by 2100	Branched from Jan 1^st^ 2015 in the historical simulation
ssp245		- Shared socioeconomic pathways (SSP2–4.5) - The medium part of the range of future forcing pathways, in which the radiative forcing reaches 4.5 W m^−2^ by 2100	
ssp585		- Shared socioeconomic pathways (SSP5-8.5) - The high end of the range of future forcing pathways, in which the radiative forcing reaches 8.5 W m^−2^ by 2100	
**Climate sensitive experiments**			
1pctCO2	150 a	- Only CO_2_ concentration is increased gradually at a rate of 1% per year - Other forcings are the same as in the piControl experiment	Branched from Jan 1^st^ 301a in piControl
abrupt-4xCO2		- CO_2_ concentration is immediately and abruptly quadrupled from the global mean 1850 value used in the piControl experiment - Other forcings are the same as in the piControl experiment	

piControl is the preindustrial control simulation and one of the DECK experiments, which represents a quasi-equilibrium state of the climate system under the imposed conditions. piControl is used to investigate the naturally occurring, unforced variability in the climate system and serves as a baseline for other experiments that branch from it. We integrated the FIO-ESM v2.0 initialized from observations for 1000 a with all forcing fields (e.g., solar radiative, greenhouse gas, and aerosols) fixed at AD 1850. The global mean of the net radiation at the top of the atmosphere averaged from 301 a to 1000 a is 0.07 W/m^2^ with a negligible linear trend of −0.0073 W/m^2^ per 100 years, which means that the energy balance of FIO-ESM v2.0 is excellent and that the climate system is stable. The trends of global mean sea temperature (0.0155 °C/100a), global mean sea salinity (−0.0001 pus/100a), and Atlantic Meridional Overturning Circulation (−0.1093 Sv/100a) from 301 a to 1000 a indicated that FIO-ESM v2.0 reached a quasi-equilibrium state after 300 a^29^. Therefore, we took the output of the last 700 years (from 301 a to 1000 a) as the piControl simulation results.

The CMIP6 historical simulation is the experiment to represent climate change over the 1850–2014 period forced by the common time-evolving forcing datasets (e.g., solar radiation, greenhouse gases, and aerosols). We integrated the historical simulation to begin in 1850, which was initialized on Jan 1^st^ for 301 a in the piControl (Fig. 2) and ended in 2014, forced by the datasets provided by CMIP6.

The future scenario experiments, which belong to the CMIP6-Endorsed Scenario Model Intercomparison Project (ScenarioMIP), are the projections of future climate change for improving understanding of the climate systems as well as future mitigation, adaptation, and impacts for climate and societal change^39^. The ScenarioMIP incorporated a new future pathway of societal development called shared socioeconomic pathways (SSPs) and designed a set of eight SSPs to provide future scenario forcings. The three future scenario experiments in this work are ssp126, ssp245, and ssp585 (Fig. 2), which represent the low, medium, and high ends of the range of future forcing pathways to produce radiative forcings of 2.6 W/m^2^, 4.5 W/m^2^, 8.5 W/m^2^ in 2100, respectively. All three experiments began in 2015, initialized on Jan 1^st^ of 2015 in the historical simulation, and ended in 2100, forced by the datasets provided by CMIP6.

The climate sensitive experiments are idealized CO_2_-forced experiments (1pctCO2 and abrupt-4xCO2) in DECK, which are used to reveal the fundamental forcing and feedback response characteristics of the models. The only externally imposed difference from piControl is the change in CO_2_ concentration. In the 1pctCO2 experiment, the CO_2_ concentration increased gradually at a rate of 1% per year. This experiment has been performed since CMIP2 and can serve as a consistent and useful benchmark for analyzing model transient climate response (TCR). In the abrupt-4xCO2 experiment, the CO_2_ concentration is immediately and abruptly quadrupled from the value in piControl. This experiment can be useful for diagnosing the fast responses of the system under an abrupt change in forcing and estimating a model’s equilibrium climate sensitivity (ECS). Both the 1pctCO2 and abrupt-4xCO2 branched from Jan 1^st^ for 301 a in piControl and integrated for 150 a (Fig. 2).

## Data Records

This dataset consists of global monthly mean and 3-hourly instantaneous wave parameters (significant wave height, mean wave direction, spectrum peak wave period, and zero-crossing wave period) from seven FIO-ESM v2.0 CMIP6 experiments, including 700-year piControl, 165-year historical, three 86-year future scenarios (ssp125, ssp245, and ssp585, respectively), and two 150-year climate sensitive simulation data (1pctCO2, and abrupt-4xCO2).

As the full dataset consists of approximately 3,000 files, it is divided into two collections. One collection stores the monthly data^40^, which are composed of 28 sub-datasets containing 52 files from four wave parameter data of 7 experiments (Online-only Table 1). The other is 3-hourly data^41^, which are composed of 24 sub-datasets containing 2892 files from four wave parameters data of 6 experiments (Online-only Table 2). The lists and conventions of these files are outlined in Online-only Tables 1 and 2.

The filenames of the monthly data are in the following format:

〈para_id〉_glob_FIO_FIO-ESM-2-0_<exp_id〉_r1i1p1f1_mon_〈year_start〉01-〈year_end〉12.nc

For the 3-hourly data, the filenames are in the following format: 〈para_id〉_glob_FIO_FIO-ESM-2-0_〈exp_id〉_r1i1p1f1_3hr_〈year_start〉0101〈hour〉00-〈year_end〉12312100.nc

where

para_id is the wave parameter (Table 3). Hs, Dm, Tp, and Tz represent the significant wave height, mean wave direction, spectrum peak wave period, and zero-crossing wave period, respectively.

**Table 3:** List of all variables in the dataset.

No.	NetCDF variable name	Description
1	time	Time for wave parameter
2	lon	Longitude for wave parameter
3	lat	Latitude for wave parameter
4	Hs	Significant wave height on the (lon, lat, time) grid
5	Dm	Mean wave direction on the (lon, lat, time) grid
6	Tp	Spectrum peak wave period on the (lon, lat, time) grid
7	Tz	Zero-crossing wave period on the (lon, lat, time) grid

exp_id represents the name of the CMIP6 experiments, including piControl, historical, ssp126, ssp245, ssp585, 1pctCO2, and abrupt-4xCO2.

year_start and year_end are represented by 4 digits, which are the beginning and end years of the file.

Hour is represented by 2 digits, where the beginning hour of the file is usually 00, except for 06, which is used for the first file of each sub-dataset.

All data files are provided in NetCDF format and are archived in the figshare digital repository^40,41^. The seven fields, including the variables of the grid information (longitude, latitude, and time) and four wave parameters in the files, are outlined in Table 3.

## Technical Validation

The MASNUM-WAM is a third-generation wavenumber spectrum wave model developed by the Key Laboratory of Marine Science and Numerical Modeling in the late 1980s^36^. MASNUM-WAM has been calibrated and adopted many times in ocean wave simulations and hindcasts, wave-current interactions, typhoons and climate simulations, and other scientific studies^37,38,42–46^. Moreover, MASNUM-WAM is now the ocean wave component of several operational ocean forecasting systems (OFS), such as the OFS for the seas off China and adjacent areas^47^, OFS for Southeast Asian Seas and OFS for the 21st-Century Maritime Silk Road^48^. Therefore, validation of the MASNUM-WAM is not shown in this study.

The validation of FIO-ESM v2.0 against observational datasets was given in Bao *et al*.^29^. The piControl results show that the global mean of the net radiation at the top of atmosphere during the last 700 years is 0.07 W/m^2^ with a negligible linear trend of −0.0073 W/m^2^ per 100 years, which indicate the energy balance of FIO-ESM v2.0 is good and the model is stable. Furthermore, they showed that FIO-ESM v2.0 could reproduce the different aspects of the climate system in global warming, surface temperatures, precipitation, and ocean circulation, etc.

ERA5^7^, one of the baseline datasets for wave climatology studies as well as providing the four wave parameters (significant wave height, mean wave direction, spectrum peak wave period, and zero-crossing wave period), was used to assess the simulation ability of wave parameters from FIO-ESM v2.0. As the aim of this dataset is to aid in wave climate research, we only represent validations for the climatology of the wave parameters in the following. The data derived from both ERA5 and FIO-ESM v2.0 were selected from 1979 to 2014 for analysis, and the monthly and 3-hourly simulated wave parameters were interpolated to the ERA5 grid at 0.5° × 0.5°.

### Monthly significant wave height, mean wave direction, spectrum peak wave period, and zero-crossing wave period

**Fig. 3:**
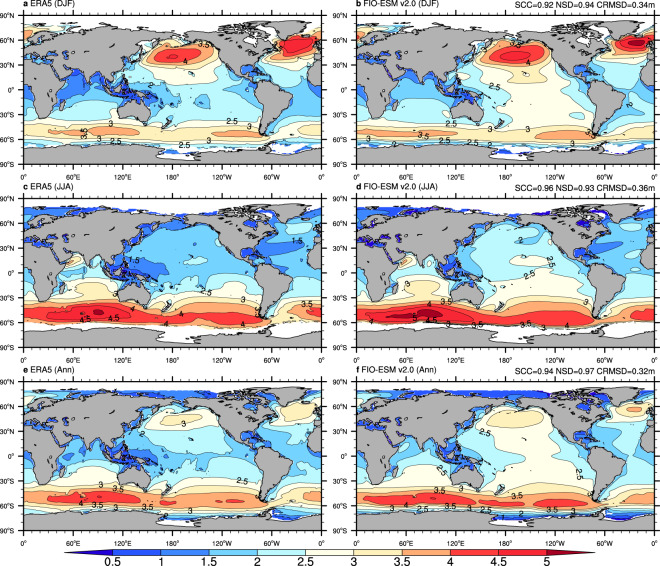
Climatological distributions of the significant wave height from monthly mean data of ERA5 (left column) and FIO-ESM v2.0 (right column). (**a**–**f**) are boreal winter (December-January-February), boreal summer (June-July-August), and annual mean results, respectively. The averaged period is from 1979 to 2014. SCC, NSD, and CRMSD represent the spatial correlation coefficient, the normalized standard deviation, and the centered-root-mean-square difference, respectively.

**Fig. 4:**
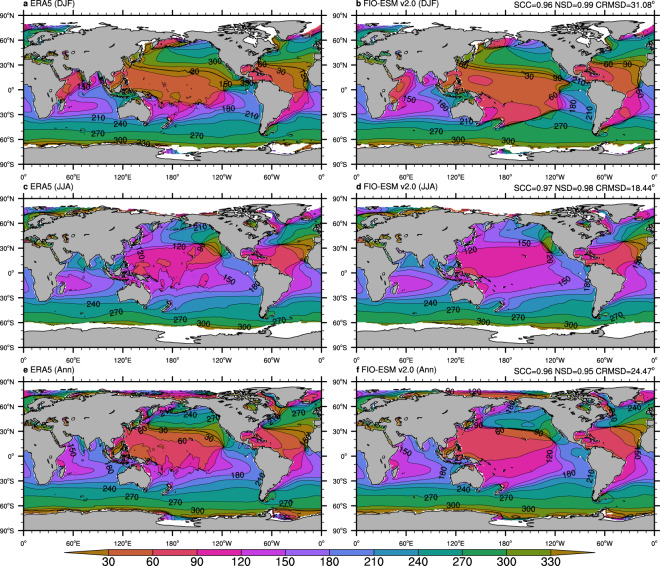
Climatological distributions of the mean wave direction from monthly mean data of ERA5 (left column) and FIO-ESM v2.0 (right column). (**a**–**f**) are boreal winter (December-January-February), boreal summer (June-July-August), and annual mean results, respectively. The averaged period is from 1979 to 2014. SCC, NSD, and CRMSD represent the spatial correlation coefficient, the normalized standard deviation, and the centered-root-mean-square difference, respectively.

**Fig. 5:**
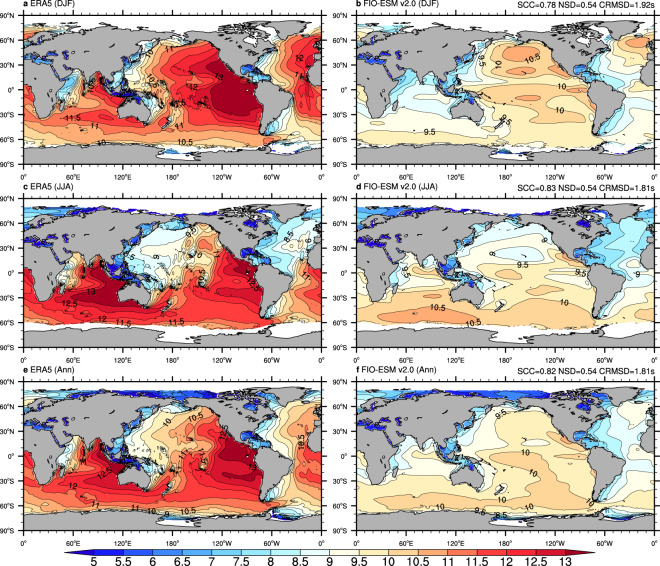
Climatological distributions of the spectrum peak wave period from monthly mean data of ERA5 (left column) and FIO-ESM v2.0 (right column). (**a**–**f**) are boreal winter (December-January-February), boreal summer (June-July-August), and annual mean results, respectively. The averaged period is from 1979 to 2014. SCC, NSD, and CRMSD represent the spatial correlation coefficient, the normalized standard deviation, and the centered-root-mean-square difference, respectively.

**Fig. 6:**
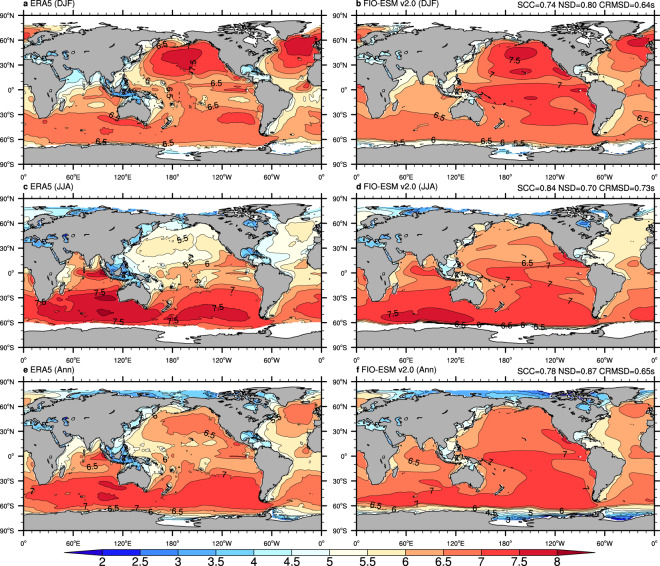
Climatological distributions of the zero-crossing wave period from monthly mean data of ERA5 (left column) and FIO-ESM v2.0 (right column). (**a**–**f**) are boreal winter (December-January-February), boreal summer (June-July-August), and annual mean results, respectively. The averaged period is from 1979 to 2014. SCC, NSD, and CRMSD represent the spatial correlation coefficient, the normalized standard deviation, and the centered-root-mean-square difference, respectively.

To assess the mean state in the spatial pattern and seasonal variation in the long-term monthly data, Figs. 3–6 show the climatological distributions of the four wave parameters (Hs, Dm, Tp, and Tz) in the boreal winter (December-January-February), boreal summer (June-July-August), and annual mean from the monthly ERA-5 and FIO-ESM v2.0 historical simulation data.

Generally, comparison against the ERA5 data in terms of annual/seasonal Hs (Fig. 3) and Dm (Fig. 4) exhibits good agreement, with the centered-root-mean-square-difference (CRMSD) values less than 0.4 m and 35°, respectively, and the spatial correlation coefficients (SCCs) values greater than 0.9 and normalized standard deviation (NSD) values close to 1. However, we found relatively less model-skill in representing the spectrum peak wave period and zero-crossing wave period. The SCCs in both annual/seasonal Tp (Fig. 5) and Tz (Fig. 6) are only approximately 0.8, while the CRMSD values are approximately 1.8 s and 0.7 s, respectively. Moreover, the model-skill in the standard deviation of Tz with NSD values greater than 0.7 is better than for Tp with NSD values less than 0.55.

Although the FIO-ESM v2.0 can capture the basic characteristics of the ocean wave, there are still several biases in spatial distributions. As shown in Fig. 3, the simulated annual mean Hs values are higher approximately 0.5 m over the North Atlantic and North Pacific oceans in summer, while the higher Hs values are simulated over the tropical Pacific Ocean throughout the year. Similar to Hs, the simulated Dm (Fig. 4), Tp (Fig. 5), and Tz (Fig. 6) also exhibit the obvious biases over the North Atlantic, North Pacific, and tropical Pacific oceans. Furthermore, the simulated Tp is less than ERA5 by approximately 1–2 s (Fig. 5), while the simulated Tz is greater by approximately 0.5–1 s (Fig. 6).

### The 3-hourly significant wave height

**Fig. 7:**
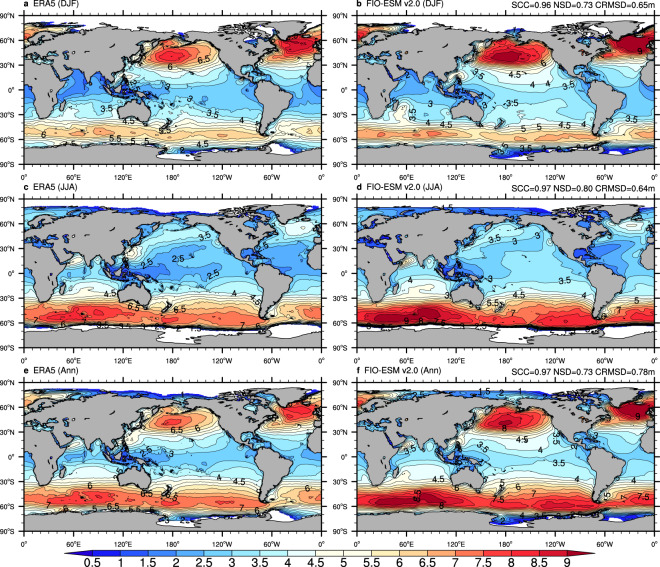
Climatological distributions of the 99-th percentile significant wave heights from 3-hourly data of ERA5 (left column) and FIO-ESM v2.0 (right column). **a** and **b**, **c** and **d**, and **e** and **f** are calculated in boreal winter (December-January-February) on a seasonal basis, boreal summer (June-July-August) on a seasonal basis, and on a yearly basis, respectively. The averaged period is 1979–2014. SCC, NSD, and CRMSD represent the spatial correlation coefficient, the normalized standard deviation, and the centered-root-mean-square difference, respectively.

The mean state has been validated above by using monthly data, so here, we focus on the extreme conditions by using 99th-percentile values of significant wave height for the 3-hourly data. Figure 7 shows the climatological distributions of the 99-th percentile (p99) significant wave height in the boreal winter (December-January-February), boreal summer (June-July-August), and whole year from the ERA5 and FIO-ESM v2.0 historical simulation data. The p99 significant wave height is calculated on a seasonal basis for boreal winter and summer and a yearly basis for the whole year.

Similar to the comparison of the monthly significant wave height between FIO-ESM v2.0 and ERA5, FIO-ESM v2.0 can reproduce the major spatial patterns and seasonal variations in the p99 significant wave height. The SCCs between ERA5 and FIO-ESM v2.0 can reach 0.96, while the CRMSD values are less than 0.8 m with the NSD values greater than 0.7. Additionally, the simulated p99 significant wave height is also greater than ERA5, particularly over the North Atlantic, North Pacific and tropical Pacific oceans.

Overall, the above analyses indicate that both the simulated spatial distributions and seasonal variations in FIO-ESM v2.0 are broadly consistent with the ERA5 data, including not only the monthly but also the 3-hourly significant wave height data. However, the simulated wave parameters still suffer several biases, especially in the North Atlantic, North Pacific, and tropical Pacific oceans, with an approximately 10% relative difference from the ERA5 data, which is similar to other ocean wave data from COWCLIP^28^.

## Usage Notes

For the 3-hourly data, as shown in Online-Only Table 2, because of one data file per year for each wave parameter, the file usually starts at 00 UTC on January 1 except for the first file of each experiment, which starts at 06 UTC. The days in February are always 28, as there is no leap year in the model.

All of the significant wave heights, mean wave directions, spectrum peak periods, and zero-crossing periods are on the native model grid named the Greenland dipole grid. The Greenland diploe grid is a latitude/longitude grid, with the North Pole displaced to Greenland to avoid singularity problems in the model. The data can be used with a wide range of postprocessing software (e.g., Ferret, NCL).

## Data Availability

The source code of FIO-ESM v2.0 will be provided upon request for the purpose of replicating the data described in this paper. The code may be requested from the corresponding author by email.

## Online-only Tables

**Online-only Table 1:** Data records in the monthly wave parameter collection (52 files).

Experiment	Wave parameters	Filename	Filename convention
piControl (28 files)	Significant wave height	Hs_glob_FIO_FIO-ESM-2-0_piControl_r1i1p1f1_mon_ < year_start > 01- < year_end > 12.nc	- 7 files per wave parameter - One data file per 100 years for each wave parameter - year_start and year_end: 4 digits; refers to the beginning and end years of the file, and comprises a 700-year section between 0301 and 1000 a.
	Mean wave direction	Dm_glob_FIO_FIO-ESM-2-0_piControl_r1i1p1f1_mon_ < year_start > 01- < year_end > 12.nc	
	Spectrum peak wave period	Tp_glob_FIO_FIO-ESM-2-0_piControl_r1i1p1f1_mon_ < year_start > 01- < year_end > 12.nc	
	Zero-crossing wave period	Tz_glob_FIO_FIO-ESM-2-0_piControl_r1i1p1f1_mon_ < year_start > 01- < year_end > 12.nc	
Historical (4 files)	Significant wave height	Hs_glob_FIO_FIO-ESM-2-0_historical_r1i1p1f1_mon_185001-201412.nc	- 1 file per wave parameter - One data file comprises a 165-year section between 1850 and 2014
	Mean wave direction	Dm_glob_FIO_FIO-ESM-2-0_historical_r1i1p1f1_mon_185001-201412.nc	
	Spectrum peak wave period	Tp_glob_FIO_FIO-ESM-2-0_piControl_r1i1p1f1_mon_185001-201412.nc	
	Zero-crossing wave period	Tz_glob_FIO_FIO-ESM-2-0_piControl_r1i1p1f1_mon_185001-201412.nc	
ssp126 (4 files)	Significant wave height	Hs_glob_FIO_FIO-ESM-2-0_ssp126_r1i1p1f1_mon_201501-210012.nc	- 1 file per wave parameter - One data file comprises an 86-year section between 2015 and 2100
	Mean wave direction	Dm_glob_FIO_FIO-ESM-2-0_ssp126_r1i1p1f1_mon_201501-210012.nc	
	Spectrum peak wave period	Tp_glob_FIO_FIO-ESM-2-0_ssp126_r1i1p1f1_mon_201501-210012.nc	
	Zero-crossing wave period	Tz_glob_FIO_FIO-ESM-2-0_ssp126_r1i1p1f1_mon_201501-210012.nc	
ssp245 (4 files)	Significant wave height	Hs_glob_FIO_FIO-ESM-2-0_ssp245_r1i1p1f1_mon_201501-210012.nc	- 1 file per wave parameter - One data file comprises an 86-year section between 2015 and 2100
	Mean wave direction	Dm_glob_FIO_FIO-ESM-2-0_ssp245_r1i1p1f1_mon_201501-210012.nc	
	Spectrum peak wave period	Tp_glob_FIO_FIO-ESM-2-0_ssp245_r1i1p1f1_mon_201501-210012.nc	
	Zero-crossing wave period	Tz_glob_FIO_FIO-ESM-2-0_ssp245_r1i1p1f1_mon_201501-210012.nc	
ssp585 (4 files)	Significant wave height	Hs_glob_FIO_FIO-ESM-2-0_ssp585_r1i1p1f1_mon_201501-210012.nc	- 1 file per wave parameter - One data file comprises an 86-year section between 2015 and 2100
	Mean wave direction	Dm_glob_FIO_FIO-ESM-2-0_ssp585_r1i1p1f1_mon_201501-210012.nc	
	Spectrum peak wave period	Tp_glob_FIO_FIO-ESM-2-0_ssp585_r1i1p1f1_mon_201501-210012.nc	
	Zero-crossing wave period	Tz_glob_FIO_FIO-ESM-2-0_ssp585_r1i1p1f1_mon_201501-210012.nc	
1pctCO2 (4 files)	Significant wave height	Hs_glob_FIO_FIO-ESM-2-0_1pctCO2_r1i1p1f1_mon_000101-015012.nc	- 1 file per wave parameter - One data file comprises a 150-year section between 0001 and 0150
	Mean wave direction	Dm_glob_FIO_FIO-ESM-2-0_1pctCO2_r1i1p1f1_mon_000101-015012.nc	
	Spectrum peak wave period	Tp_glob_FIO_FIO-ESM-2-0_1pctCO2_r1i1p1f1_mon_000101-015012.nc	
	Zero-crossing wave period	Tz_glob_FIO_FIO-ESM-2-0_1pctCO2_r1i1p1f1_mon_000101-015012.nc	
abrupt-4xCO2 (4 files)	Significant wave height	Hs_glob_FIO_FIO-ESM-2-0_abrupt-4xCO2_r1i1p1f1_mon_000101-015012.nc	- 1 file per wave parameter - One data file comprises a 150-year section between 0001 and 0150
	Mean wave direction	Dm_glob_FIO_FIO-ESM-2-0_abrupt-4xCO2_r1i1p1f1_mon_000101-015012.nc	
	Spectrum peak wave period	Tp_glob_FIO_FIO-ESM-2-0_abrupt-4xCO2_r1i1p1f1_mon_000101-015012.nc	
	Zero-crossing wave period	Tz_glob_FIO_FIO-ESM-2-0_abrupt-4xCO2_r1i1p1f1_mon_000101-015012.nc	

**Online-only Table 2:** Data records in 3-hourly wave parameter collection (2892 files).

Experiment	Wave parameters	Filename	Filename convention
Historical (660 files)	Significant wave height	Hs_glob_FIO_FIO-ESM-2-0_historical_r1i1p1f1_3hr_ < year_start > 0101 < hour > 00- < year_end > 12312100.nc	- 165 files per wave parameter - One data file per year for each wave parameter - year_start and year_end: 4 digits; refers to the beginning and end years of the file and comprises a 165-year section between 1850 and 2014 - hour: 2 digits; 00, except for 06 for the first file - No leap year
	Mean wave direction	Dm_glob_FIO_FIO-ESM-2-0_historical_r1i1p1f1_3hr_ < year_start > 0101 < hour > 00- < year_end > 12312100.nc	
	Spectrum peak wave period	Tp_glob_FIO_FIO-ESM-2-0_piControl_r1i1p1f1_3hr_ < year_start > 0101 < hour > 00- < year_end > 12312100.nc	
	Zero-crossing wave period	Tz_glob_FIO_FIO-ESM-2-0_piControl_r1i1p1f1_3hr_ < year_start > 0101 < hour > 00- < year_end > 12312100.nc	
ssp126 (344 files)	Significant wave height	Hs_glob_FIO_FIO-ESM-2-0_ssp126_r1i1p1f1_3hr_ < year_start > 0101 < hour > 00- < year_end > 12312100.nc	- 86 files per wave parameter - One data file per year for each wave parameter - year_start and year_end: 4 digits; refers to the beginning and end years of the file and comprises an 86-year section between 2015 and 2100 - hour: 2 digits; 00, except for 06 for the first file - No leap year
	Mean wave direction	Dm_glob_FIO_FIO-ESM-2-0_ssp126_r1i1p1f1_3hr_ < year_start > 0101 < hour > 00- < year_end > 12312100.nc	
	Spectrum peak wave period	Tp_glob_FIO_FIO-ESM-2-0_ssp126_r1i1p1f1_3hr_ < year_start > 0101 < hour > 00- < year_end > 12312100.nc	
	Zero-crossing wave period	Tz_glob_FIO_FIO-ESM-2-0_ssp126_r1i1p1f1_3hr_ < year_start > 0101 < hour > 00- < year_end > 12312100.nc	
ssp245 (344 files)	Significant wave height	Hs_glob_FIO_FIO-ESM-2-0_ssp245_r1i1p1f1_3hr_ < year_start > 0101 < hour > 00- < year_end > 12312100.nc	- 86 files per wave parameter - One data file per year for each wave parameter - year_start and year_end: 4 digits; refers to the beginning and end years of the file and comprises an 86-year section between 2015 and 2100 - hour: 2 digits; 00, except for 06 for the first file - No leap year
	Mean wave direction	Dm_glob_FIO_FIO-ESM-2-0_ssp245_r1i1p1f1_3hr_ < year_start > 0101 < hour > 00- < year_end > 12312100.nc	
	Spectrum peak wave period	Tp_glob_FIO_FIO-ESM-2-0_ssp245_r1i1p1f1_3hr_ < year_start > 0101 < hour > 00- < year_end > 12312100.nc	
	Zero-crossing wave period	Tz_glob_FIO_FIO-ESM-2-0_ssp245_r1i1p1f1_3hr_ < year_start > 0101 < hour > 00- < year_end > 12312100.nc	
ssp585 (344 files)	Significant wave height	Hs_glob_FIO_FIO-ESM-2-0_ssp585_r1i1p1f1_3hr_ < year_start > 0101 < hour > 00- < year_end > 12312100.nc	- 86 files per wave parameter - One data file per year for each wave parameter - year_start and year_end: 4 digits; refers to the beginning and end years of the file and comprises an 86-year section between 2015 and 2100 - hour: 2 digits; 00, except for 06 for the first file - No leap year
	Mean wave direction	Dm_glob_FIO_FIO-ESM-2-0_ssp585_r1i1p1f1_3hr_ < year_start > 0101 < hour > 00- < year_end > 12312100.nc	
	Spectrum peak wave period	Tp_glob_FIO_FIO-ESM-2-0_ssp585_r1i1p1f1_3hr_ < year_start > 0101 < hour > 00- < year_end > 12312100.nc	
	Zero-crossing wave period	Tz_glob_FIO_FIO-ESM-2-0_ssp585_r1i1p1f1_3hr_ < year_start > 0101 < hour > 00- < year_end > 12312100.nc	
1pctCO2 (600 files)	Significant wave height	Hs_glob_FIO_FIO-ESM-2-0_1pctCO2_r1i1p1f1_3hr_ < year_start > 0101 < hour > 00- < year_end > 12312100.nc	- 150 files per wave parameter - One data file per year for each wave parameter - year_start and year_end: 4 digits; refers to the beginning and end years of the file and comprises a 150-year section between 0001 and 0150 - hour: 2 digits; 00, except for 06 for the first file - No leap year
	Mean wave direction	Dm_glob_FIO_FIO-ESM-2-0_1pctCO2_r1i1p1f1_3hr_ < year_start > 0101 < hour > 00- < year_end > 12312100.nc	
	Spectrum peak wave period	Tp_glob_FIO_FIO-ESM-2-0_1pctCO2_r1i1p1f1_3hr_ < year_start > 0101 < hour > 00- < year_end > 12312100.nc	
	Zero-crossing wave period	Tz_glob_FIO_FIO-ESM-2-0_1pctCO2_r1i1p1f1_3hr_ < year_start > 0101 < hour > 00- < year_end > 12312100.nc	
abrupt-4xCO2 (600 files)	Significant wave height	Hs_glob_FIO_FIO-ESM-2-0_abrupt-4xCO2_r1i1p1f1_3hr_ < year_start > 0101 < hour > 00- < year_end > 12312100.nc	- 150 files per wave parameter - One data file per year for each wave parameter - year_start and year_end: 4 digits; refers to the beginning and end years of the file and comprises a 150-year section between 0001 and 0150 - hour: 2 digits; 00, except for 06 for the first file - No leap year
	Mean wave direction	Dm_glob_FIO_FIO-ESM-2-0_abrupt-4xCO2_r1i1p1f1_3hr_ < year_start > 0101 < hour > 00- < year_end > 12312100.nc	
	Spectrum peak wave period	Tp_glob_FIO_FIO-ESM-2-0_abrupt-4xCO2_r1i1p1f1_3hr_ < year_start > 0101 < hour > 00- < year_end > 12312100.nc	
	Zero-crossing wave period	Tz_glob_FIO_FIO-ESM-2-0_abrupt-4xCO2_r1i1p1f1_3hr_ < year_start > 0101 < hour > 00- < year_end > 12312100.nc	
